# RNA-Seq analysis of early transcriptional responses to activation in the leukaemic Jurkat E6.1 T cell line

**DOI:** 10.12688/wellcomeopenres.15748.1

**Published:** 2020-03-03

**Authors:** Suet Ling Felce, Gillian Farnie, Michael L. Dustin, James H. Felce

**Affiliations:** 1 Structural Genomics Consortium, Botnar Research Centre, NDORMS, University of Oxford, Oxford, OX3 7LD, UK; 2 Kennedy Institute of Rheumatology, University of Oxford, Oxford, OX3 7FY, UK

**Keywords:** RNA-Seq, T cell activation, Jurkats, Chemokines

## Abstract

**Background:** The leukaemia-derived Jurkat E6.1 cell line has been used as a model T cell in the study of many aspects of T cell biology, most notably activation in response to T cell receptor (TCR) engagement.

**Methods:** We present whole-transcriptome RNA-Sequencing data for Jurkat E6.1 cells in the resting state and two hours post-activation via TCR and CD28. We compare early transcriptional responses in the presence and absence of the chemokines CXCL12 and CCL19, and perform a basic comparison between observed transcriptional responses in Jurkat E6.1 cells and those in primary human T cells using publicly deposited data.

**Results:** Jurkat E6.1 cells have many of the hallmarks of standard T cell transcriptional responses to activation, but lack most of the depth of responses in primary cells.

**Conclusions:** These data indicate that Jurkat E6.1 cells hence represent only a highly simplified model of early T cell transcriptional responses.

## Introduction

Adaptive immunity is centred on the clonal selection and activation of lymphocytes, most importantly T cells, which provide stimulation and regulation to other cells of the immune system as well as directly killing infected cells. T cells become activated in response to binding of their clonally specific T cell receptors (TCRs) to cognate peptide-major histocompatibility complexes (pMHCs) on the surface of interacting antigen-presenting cells. This leads to recruitment and phosphorylation of a series of kinases and adaptor proteins to the TCR, intracellular Ca ^2+^ mobilisation, and consequent downstream changes in gene expression – largely due to activation of nuclear factor of activated T cells (NFAT) – which mark the transition to a fully activated state. Much of our understanding of the early events of T cell activation has been facilitated by the use of a small number of T cell lines, due largely to their relative ease of handling, manipulation, and transfection. Arguably the most widely used such case is the Jurkat cell line, which was originally isolated from the peripheral leukaemic T cells of a 14-year-old boy in the late 1970s ( Abraham & Weiss, 2004). The E6.1 clone was subsequently derived from this due to its high capacity for interleukin-2 (IL2) secretion, and rapidly became the standard line for many prominent T cell biologists at the time.

The contribution of the Jurkat E6.1 line to T cell research is unquestionable and it is still widely used despite greatly improved technologies for *ex vivo* manipulation of primary T cells. Nonetheless, it is well described that some significant differences exist between the activatory processes and outcomes in primary and Jurkat T cells; an unavoidable consequence of the abnormal origins of the line. For example, Jurkats exhibit elevated phosphorylation of several core signalling proteins, such as phospholipase C γ1 (PLCγ1) and extracellular signal-related kinases (ERKs) 1 and 2 ( Bartelt *et al.,* 2009). This is linked in part to constitutive phosphatidylinositol 3-kinase (PI3K) activity due to a defect in phosphatase and tensin homolog (PTEN) expression ( Shan *et al.,* 2000), which also leads to hypersensitivity to TCR engagement compared to primary T cells. Relative to primary cells, Jurkats also exhibit differences in their cytoskeletal dynamics and organisation ( Colin-York *et al.,* 2019), which may influence their response to activation due to effects on the formation of the immunological synapse ( Dustin, 2014). Hence, although Jurkats have retained many of the core aspects of normal T cell signalling, there are many features of their biology that deviate substantially from primary cells.

One complex aspect of T cell activation is how signals deriving from the TCR become integrated with those from other modulatory receptors. An interesting example of this is the contribution of chemokines to T cell activation, given that chemokines are more usually associated with cell migration ( Hughes & Nibbs, 2018) and their effects on activation are not always considered. Nonetheless, many reports exist of chemokine receptors ligation modulating T cell responses to activation through the TCR; most prominently CXCR4 (e.g. Kumar *et al.,* 2006; Molon *et al.,* 2005), CCR7 (e.g. Gollmer *et al.,* 2009; Laufer *et al.,* 2019), and CXCR3 ( Dar & Knechtle, 2007; Newton *et al.,* 2009). It is not known how such effects may influence the early transcriptional responses to activation in Jurkats, and hence if Jurkats would be a suitable model for the interrogation of such processes.

Here, we present whole transcriptome RNA-Sequencing (RNA-Seq) data for Jurkat E6.1 cells under resting conditions and two hours post-activation in the presence and absence of the chemokines CXCL12 and CCL19, which ligate the established costimulatory receptors CXCR4 and CCR7, respectively. In order to probe the differences in early transcriptional responses between Jurkats and primary T cells, we compare our data to publicly deposited data derived from equivalently activated primary T cells.

## Methods

### Jurkat E6.1 culture and stimulation

Jurkat E6.1 cells were cultured in RPMI-1640 supplemented with 10% FCS (Gibco), 4 mM L-glutamine (Gibco), 10 mM HEPES (Gibco), 1 mM sodium pyruvate (Gibco), and 1% penicillin-streptomycin solution (Gibco) at 37°C, 5% CO _2_. Cells were passaged every ~3 days to remain at ~1×10 ^6^/ml. Routine mycoplasma testing was performed using the PlasmoTest™ Mycoplasma Detection Kit (InvivoGen) as per the manufacturer’s instructions.

Stimulation was performed using anti-human CD3/CD28 Dynabeads (Gibco) in complete growth medium. Cells were centrifuged at 300 × g for 5 min then resuspended in warm growth medium at 2.5 × 10 ^6^/ml. Next, 2 ml/well of cell suspension was added to 3 wells of a 6-well plate and equilibrated at 37°C, 5% CO _2_ for 1 h. Following this, 2 × 10 ^7^ anti-CD3/CD28 beads were washed with growth medium and resuspended in 200 μl growth medium. Then, 100 μl of beads were then added to 2 wells and gently mixed, the other well was left as the resting condition. After 5 min, CXCL12 (PeproTech) and CCL19 (PeproTech) were added to one well to a final concentration of 100 ng/ml each. Cells were returned to the incubator for 2 h (for RNA-Seq experiments) or 2, 4, 6, or 24 h (for flow cytometry experiments), before removal and downstream use.

### RNA isolation and RNA-Seq

For each condition, total RNA was isolated from 5 × 10 ^6^ cells, which were centrifuged at 300 × g for 5 min then resuspended in 2 ml TRIzol® reagent (ThermoFisher), incubated at room temperature for 10 min before adding 0.4 ml chloroform. The cell suspension was mixed thoroughly then centrifuged at 5,000 × g, 4°C for 30 min. The top aqueous layer (~800 μl) was carefully removed and replaced with an equivalent volume of isopropanol. Samples were then centrifuged at 17,000 × g, 4°C for 30 min to pellet the total RNA. Isopropanol was removed and the pellet washed with 1 ml EtOH, then centrifuged again at 17,000 × g, 4°C for 5 min. EtOH was removed and the pellet was air-dried for 15 min then resuspended in 200 μl RNase-free H _2_O. To remove contaminant DNA, 22 μl 10× TURBO DNase buffer (ThermoFisher) and 2 μl (4U) TURBO DNase (ThermoFisher) were added the RNA suspension then incubated at 37°C for 30 min. The described TRIzol®-chloroform extraction protocol was then repeated to remove the DNase, and the final RNA sample resuspended in 200 μl RNase-free H _2_O. RNA was isolated twice for each condition in two independent experiments.

ds-cDNA libraries for each sample were prepared and sequenced at the Wellcome Trust Centre for Human Genetics, Oxford, using the HiSeq ^®^ 4000 Sequencing System (Illumina).

### RNA-Seq data analysis

Individual sequences were aligned to the human GRCh37.EBVB95-8wt.ERCC reference genome and quantified using HISAT2 ( Kim *et al.,* 2019), version 2.0.4. Differentially expressed genes were determined using DESeq2 ( Love *et al.,* 2014), version 1.22.2, ± 1 log2 fold and FDR adjusted p-value p<0.05. Differentially expressed gene lists were generated for multiple datasets as indicated in the results. These were between: (i) resting vs. 2 h stimulated Jurkats; (ii) resting vs. 2 h stimulated Jurkats + chemokines; (iii) 2 h stimulated Jurkats vs. 2 h stimulated Jurkats + chemokines; (iv) resting vs. 2 h stimulated primary memory CD4 ^+^ T cells (NCBI SRA: SRP026389); (v) resting vs. 24 h stimulated primary memory CD4 ^+^ T cells (NCBI SRA: SRP026389); and (vi) resting vs. 24 h stimulated total primary CD4 ^+^ T cells (GEO: GSE122735).

Comparisons of individual differentially expressed gene lists were carried out using vennCounts as part of the limma package ( Ritchie *et al.,* 2015), version 3.38.3. Gene Ontology enrichment analysis of individual and shared/non-shared gene sets was carried out using the enrichGO function from clusterProfiler ( Yu *et al.,* 2012), version 3.10.1.

### Primary CD4 ^+^ T cell isolation and culture

Primary human CD4 ^+^ T cells were isolated using the RosetteSep Human CD4 ^+^ T Cell Enrichment Cocktail (StemCell Technologies) as per the manufacturer’s instructions from leukocyte cones provided by UK National Health Service Blood and Transplant. Isolated cells were cultured in RPMI-1640 supplemented with 10% FCS (Gibco), 4 mM L-glutamine (Gibco), 10 mM HEPES (Gibco), 1% non-essential amino acid solution (Gibco), and 1% penicillin-streptomycin solution (Gibco) at 37°C, 5% CO _2_ for between 24 and 72 h before stimulating in the same manner as for Jurkat cells.

### Flow cytometry

Following stimulation for flow cytometry experiments, cells were fixed with 4% para-formaldehyde for 10 min before being washed 3 times with PBS and blocked/quenched with 5% bovine serum + 0.1 mM glycine overnight at 4°C. Cells were stained with 1 μg/ml of anti-BTLA AlexaFluor 647 (RRID AB_2650979; BioLegend Cat. No. 344519), anti-CTLA4 PE (RRID AB_10645522; BioLegend Cat. No. 349905), anti-CD86 Brilliant Violet 421 (RRID AB_10899582; BioLegend Cat. No. 305425), anti-CD69 APC (RRID AB_314844; BioLegend Cat. No. 310909), or anti-CD25 FITC (RRID AB_314273; BioLegend Cat. No. 302603) for 1 h at room temperature then washed 3 times with PBS before being analysed using a FACSCanto II™ flow cytometer (BD Biosciences). Data were analysed using FlowJo version 8.8.7. Staining was performed in three independent experiments with cells from different donors.

### Statistical analysis

The prcomp function was used for the principal component analysis and plotPCA was used for principal component analysis and visualisation; both are part of DESeq2 package. Intensity values derived from flow cytometry data were analysed and visualised using GraphPad Prism, version 8.2.1.

## Results

### Jurkat responses broadly correspond to expected effects of T cell activation and are unaffected by the presence of chemokines

RNA-Seq was performed on total mRNA collected from Jurkat E6.1 cells under both resting and activated conditions (total mapped counted given in *Extended data* - Dataset S1 ( Felce, 2020b)). Activation was performed using anti-CD3, anti-CD28 beads in the absence of exogenous IL2, and with either 0 or 100 ng/ml soluble CXCL12 and CCL19. Cells were activated for 2 h before lysis and mRNA extraction. This time point was used in order to interrogate only early transcriptional responses that occur during the expected lifetime of the T cell immunological synapse (up to the order of several hours) and hence may influence events occurring within the contact. Sequencing yielded between 4x10 ^7^ and 4.9x10 ^7^ individual reads per sample, and were performed as biological replicates with duplicate mRNA samples isolated in independent experiments. Following mapping to the human GRCh37.EBVB95-8wt.ERCC reference genome, differential gene expression was determined using DESeq2. Multimapped reads were not included in downstream analysis.

**Figure 1.**
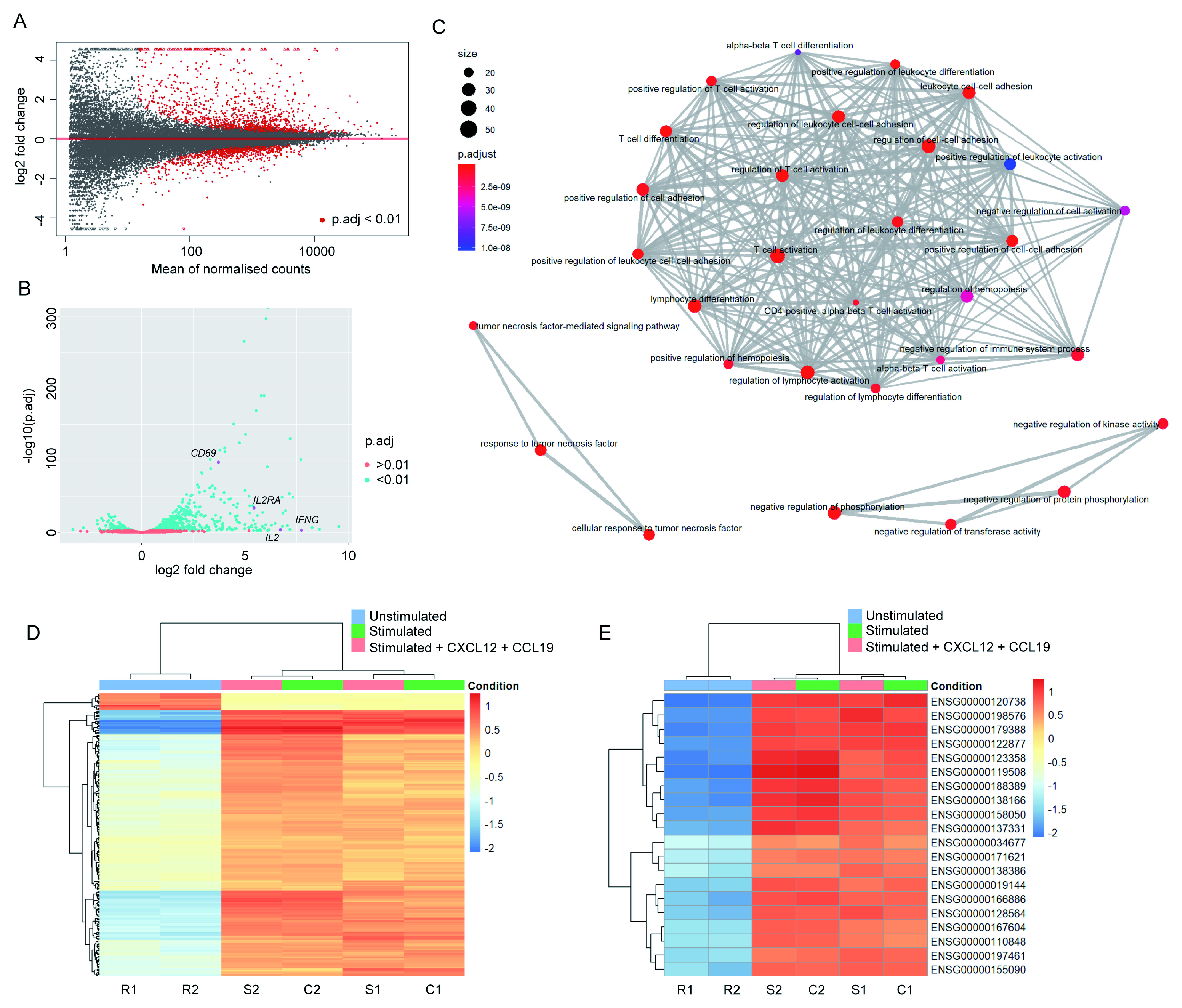
Changes in RNA abundance upon Jurkat stimulation broadly correspond to expected effects of T cell activation. ( **A**) Mean average plot for resting vs. 2 h stimulated Jurkats. Normalised counts are given as mean across both samples. All mapped genes are displayed – those with a false discovery rate (p.adj) below 0.01 are shown in red. ( **B**) Volcano plot for resting vs. 2 h stimulated Jurkats. Most significantly altered genes undergo upregulation, including many typical markers of T cell activation (indicated in magenta). ( **C**) Enrichment map plot of Gene Ontology terms significantly enriched among differentially expressed genes in activated vs. resting Jurkats. Nodes are coloured according to false discovery rate, and sized according to number of associated genes. As expected, the primary cluster is associated with terms linked to T cell activation. ( **D**) Heatmap of 200 most significantly expressed genes across all samples. Genes are coloured according to the absolute difference between log2-transformed raw read count and the other samples in the same row. Dendrograms indicate hierarchical clustering of gene (left) or sample (top) similarities. Samples R1 and R2 indicate replicate samples for resting cells; S1 and S2, stimulated; C1 and C2, stimulated + chemokines. Samples stimulated in the presence and absence of chemokines do not cluster differentially from one another, indicating no significant effect. ( **E**) Heatmap of 20 genes with highest variance across samples, coloured as in ( **D**).

**Figure 2.**
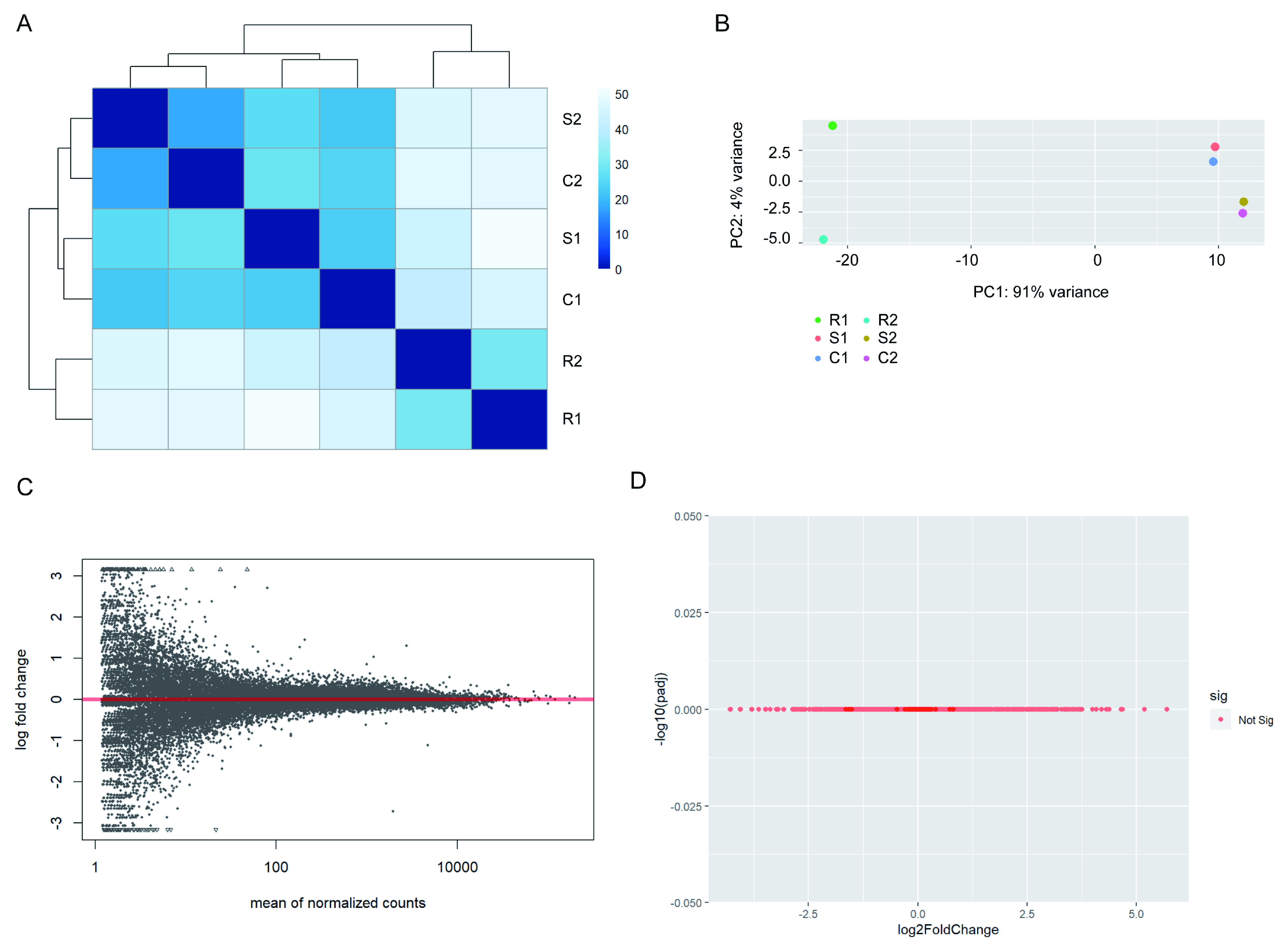
Stimulated Jurkat samples exhibit no significant differences. ( **A**) Heatmap of sample-to-sample distances for all Jurkat samples. Stimulated samples in the presence and absence of chemokines do not form discrete clusters. ( **B**) Principal component analysis plot of all Jurkat samples. ( **C**) Mean average plot for stimulated vs. stimulated + chemokine Jurkats. All mapped genes are displayed – none exhibited a false discovery rate below 0.01. ( **B**) Volcano plot for stimulated vs. stimulated + chemokine Jurkats.

Firstly, gene expression was compared between resting Jurkats and those activated in the absence of chemokines. As expected, expression levels of many genes with a wide range of mean read counts were substantially altered in response to cellular activation ( Figure 1A; full differential gene list given in *Extended data* - Dataset S2 ( Felce, 2020b)). The majority of genes with significantly (p.adj<0.01) altered expression upon activation underwent upregulation, including numerous well-characterised markers of T cell activation, e.g. *CD69*, *IL2*, *IL2RA*, and *IFNG* ( Figure 1B). GO enrichment analysis revealed that GO terms associated with significantly differentially expressed genes were largely connected to T cell activation, differentiation, and/or adhesion ( Figure 1C; *Extended data* - Dataset S3 ( Felce, 2020b)), as expected. The same comparison performed between resting Jurkats and those activated in the presence of soluble chemokines to receptors CXCR4 (CXCL12) and CCR7 (CCL19) yielded comparable results, with no apparent effect of chemokines on differential gene expression in response to activation ( Figure 1D, E). Accordingly, when sample-to-sample and principal component variances are compared all samples within activated conditions cluster together due to high similarity, and are clearly distinct from samples in resting state ( Figure 2A, B). Direct differential gene expression analysis between the activated samples in the presence and absence of chemokine yielded no significantly different genes ( Figure 2C, D).

### The majority of early transcriptional responses observed in primary T cells are not replicated in Jurkats

Whilst the observed early transcriptional responses in Jurkats unquestionably correlate with known processes of T cell activation, we were interested in whether they deviated substantially from the situation in primary cells. We therefore took the opportunity to compare our dataset with publicly deposited RNA-Seq data of primary T cell activation. At present, no data are publicly available for primary human CD4 ^+^ T cells activated in an analogous manner for 2 h; however, a comparable analysis of activated primary CCR6 ^+^ CD4 ^+^ memory T cells has been previously published ( Zhao *et al.,* 2014). In this case the activatory conditions included Th17-polarising cytokines and antibodies, which were not present during the stimulation of Jurkats and hence may skew transcriptional responses accordingly. Nonetheless, this dataset allowed for a crude comparison between Jurkat and primary cells.

**Figure 3.**
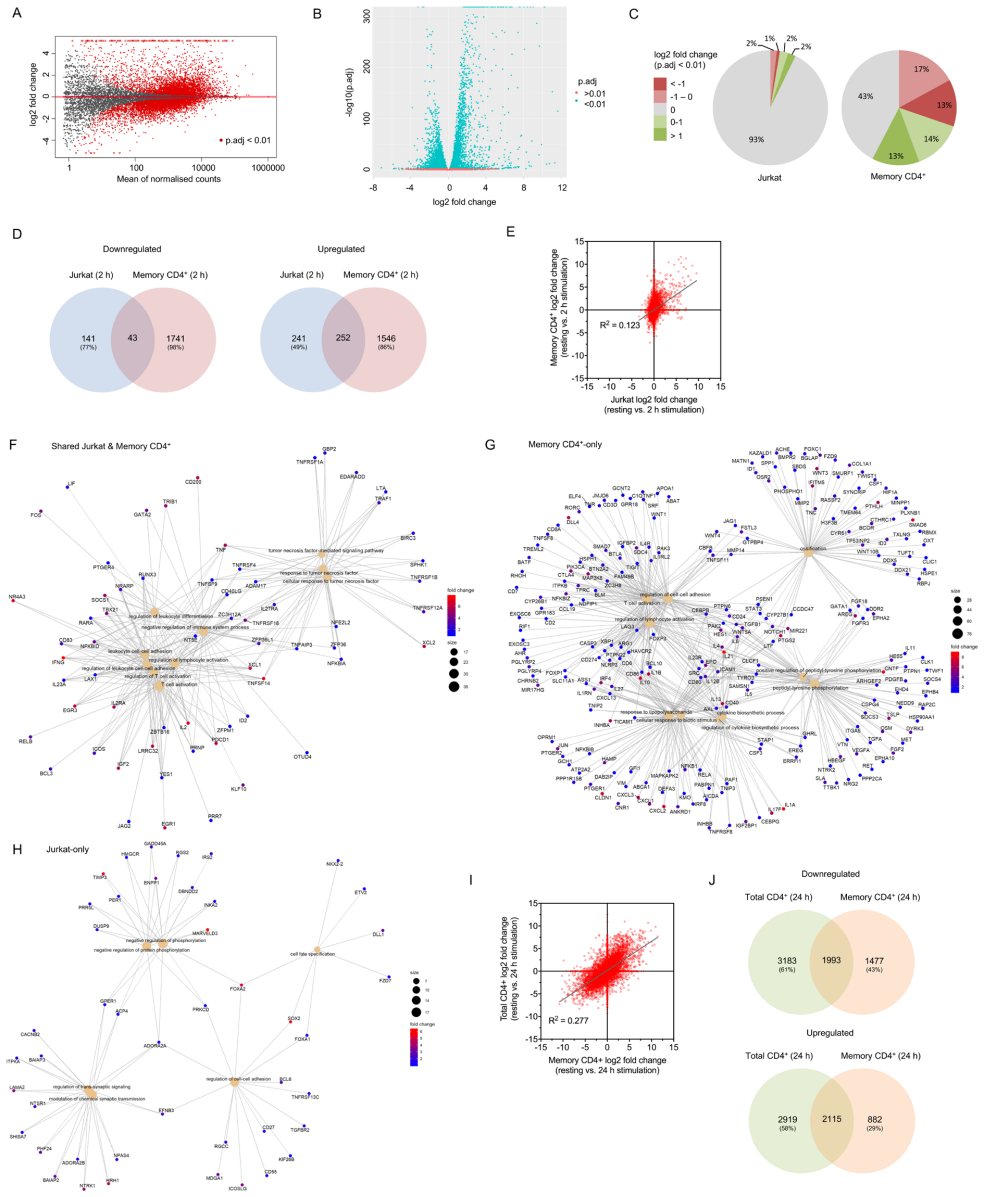
Activation-induced changes to Jurkat RNA expression represent only a minority of those in primary T cells. ( **A**) Mean average plot for resting vs. 2 h stimulated primary memory CD4 ^+^ T cells. All mapped genes are displayed – those with a false discovery rate below 0.01 are shown in red. ( **B**) Volcano plot for resting vs. 2 h stimulated primary memory CD4 ^+^ T cells. ( **C**) Proportion of upregulated or downregulated significantly differentially expressed genes in Jurkats and primary memory CD4+ T cells activated for 2 h. ( **D**) Venn diagrams of total upregulated (log2 fold change > 1) and downregulated (log2 fold change < -1) genes shared and non-shared between Jurkats and primary memory CD4 ^+^ T cells. ( **E**) Log2 fold change for all genes across resting and 2 h activated conditions in Jurkats vs *.* primary memory CD4 ^+^ cells. ( **F**– **H**) Gene concept network plots of upregulated genes associated with up to 10 most significant GO terms in both Jurkats and primary memory CD4 ^+^ cells ( **F**) or in just one cell type ( **G** & **H**). Only 6 significant terms were identified for Jurkat-only genes. Terms associated with T cell activation are enriched in both shared and memory CD4 ^+^-only sets, but not Jurkat-only sets. ( **I**) Log2 fold change for all genes across resting and 24 h activated conditions in primary memory CD4 ^+^ vs. total CD4 ^+^ T cells. ( **J**) Venn diagrams of total upregulated (log2 fold change > 1) and downregulated (log2 fold change < -1) genes shared and non-shared between primary memory CD4 ^+^ and total CD4 ^+^ T cells.

We examined unstimulated and 2 h stimulated raw read counts derived from the publicly deposited dataset (NCBI SRA: SRP026389) using the same analysis as for the Jurkat-derived data, then compared log2 fold change for all genes across both cell types and the fraction of significantly up- or downregulated genes. A far greater proportion of genes expressed in primary memory CD4 ^+^ cells (57%) had significantly altered expression upon stimulation compared to those in Jurkats (7%; Figure 3). Of those genes differentially expressed in Jurkats, 51% of upregulated and 33% of downregulated genes were similarly regulated in primary cells; however, the vast majority (86% and 98% of up- and downregulated genes, respectively) of genes with altered expression in primary cells exhibited no substantial change in Jurkats ( Figure 3, C). As would be expected, genes that underwent upregulation in both cell types were strongly associated with GO terms linked to T cell activation, cell-cell adhesion, cytokine regulation, and other related biological processes ( Figure 3, Table 1; *Extended data* - Dataset S4 ( Felce, 2020b)). Genes upregulated only in primary cells also strongly correlated with many such processes ( Figure 3, Table 1; *Extended data* - Dataset S4 ( Felce, 2020b)), suggesting that Jurkats lack either expression or early activation-induced regulation of many genes associated with normal T cell responses. Examples of such genes span a wide range of functions, including inhibitory surface receptors (e.g. *BTLA*, *CTLA4*, *TIGIT*, *LAG3*), regulatory ligands (e.g. *CD40*, *CD80*, *CD86*, *PDL1*), adhesion molecules (e.g. *ICAM1*, *CD2*, *CD58*), cytokines (e.g. *TGF1B*, *IL4*, *IL6*, *IL10*), chemokines (e.g. *CCL19*, *CXCL1*, *CXCL2*, *CXCL3*), G protein-coupled receptors (e.g. *CNR1*, *PTGER1*, *GPR183*, *GPR18*), and transcription factors ( *e.g. FOXP1*, *FOXP3*, *IRF8*, *CBFB*). The relatively small number of genes upregulated in Jurkats but not primary cells exhibited weak association to six biological processes related to the regulation of protein phosphorylation or synaptic transmission ( Figure 3H, Table 1; *Extended data* - Dataset S4 ( Felce, 2020b)). Shared and non-shared downregulated genes across both cell types did not generally correlate significantly with known biological processes, except in the case of primary-only downregulated genes, which included a number of Toll-like receptors and associated proteins ( *Extended data* - Dataset S4 ( Felce, 2020b)).

**Table 1.** Gene Ontology (GO) terms associated with significantly upregulated genes in stimulated Jurkat and primary T cells. Top 20 (6 for Jurkat) most significant GO terms associated with genes upregulated in Jurkat, primary memory CD4 ^+^ T cells, or both stimulated for 2 h. Redundant terms have been removed for clarity. A full list of significant terms is available in the Extended Data (Dataset S4).

Cell types	GO Term	Description	P-value
Memory CD4+ and Jurkat (2 h)	GO:0042110	T cell activation	6.79E-15
	GO:1903037	Regulation of leukocyte cell-cell adhesion	1.63E-12
	GO:0071356	Cellular response to tumor necrosis factor	3.37E-12
	GO:1902105	Regulation of leukocyte differentiation	4.86E-12
	GO:0002683	Negative regulation of immune system process	2.51E-11
	GO:0001818	Negative regulation of cytokine production	2.90E-09
	GO:0001819	Positive regulation of cytokine production	5.60E-07
	GO:0032496	Response to lipopolysaccharide	6.92E-07
	GO:0051348	Negative regulation of transferase activity	1.40E-06
	GO:0071901	Negative regulation of protein serine/threonine kinase activity	1.42E-06
	GO:0045444	Fat cell differentiation	1.65E-06
	GO:0001503	Ossification	6.61E-06
	GO:0032609	Interferon-gamma production	6.61E-06
	GO:1904035	Regulation of epithelial cell apoptotic process	7.33E-06
	GO:0097191	Extrinsic apoptotic signaling pathway	1.16E-05
	GO:1901652	Response to peptide	1.54E-05
	GO:0051090	Regulation of DNA binding transcription factor activity	2.03E-05
	GO:0051051	Negative regulation of transport	2.35E-05
	GO:0002699	Positive regulation of immune effector process	2.62E-05
	GO:0060759	Regulation of response to cytokine stimulus	6.39E-05
Memory CD4+ only (2 h)	GO:0032496	Response to lipopolysaccharide	2.53E-10
	GO:0071216	Cellular response to biotic stimulus	4.02E-09
	GO:0050731	Positive regulation of peptidyl-tyrosine phosphorylation	4.02E-09
	GO:0042110	T cell activation	1.07E-08
	GO:0022407	Regulation of cell-cell adhesion	3.92E-08
	GO:0001503	Ossification	3.92E-08
	GO:0042035	Regulation of cytokine biosynthetic process	4.91E-08
	GO:0030099	Myeloid cell differentiation	3.91E-07
	GO:1902895	Positive regulation of pri-miRNA transcription by RNA polymerase II	3.45E-06
	GO:0002822	Regulation of adaptive immune response based on somatic recombination of immune receptors	3.52E-06
	GO:0032944	Regulation of mononuclear cell proliferation	3.52E-06
	GO:0070555	Response to interleukin-1	3.52E-06
	GO:1902105	Regulation of leukocyte differentiation	9.17E-06
	GO:0042254	Ribosome biogenesis	1.87E-05
	GO:0090287	Regulation of cellular response to growth factor stimulus	1.87E-05
	GO:0001818	Negative regulation of cytokine production	1.87E-05
	GO:0007178	Transmembrane receptor protein serine/threonine kinase signaling pathway	2.67E-05
	GO:0002699	Positive regulation of immune effector process	2.71E-05
	GO:0016074	snoRNA metabolic process	2.71E-05
	GO:0048608	Reproductive structure development	3.38E-05
Jurkat only (2 h)	GO:0042326	Negative regulation of phosphorylation	0.004101547
	GO:0050804	Modulation of chemical synaptic transmission	0.004101547
	GO:0099177	Regulation of trans-synaptic signaling	0.004101547
	GO:0022407	Regulation of cell-cell adhesion	0.005367298
	GO:0001933	Negative regulation of protein phosphorylation	0.007798348
	GO:0001708	Cell fate specification	0.0094938

**Figure 4.**
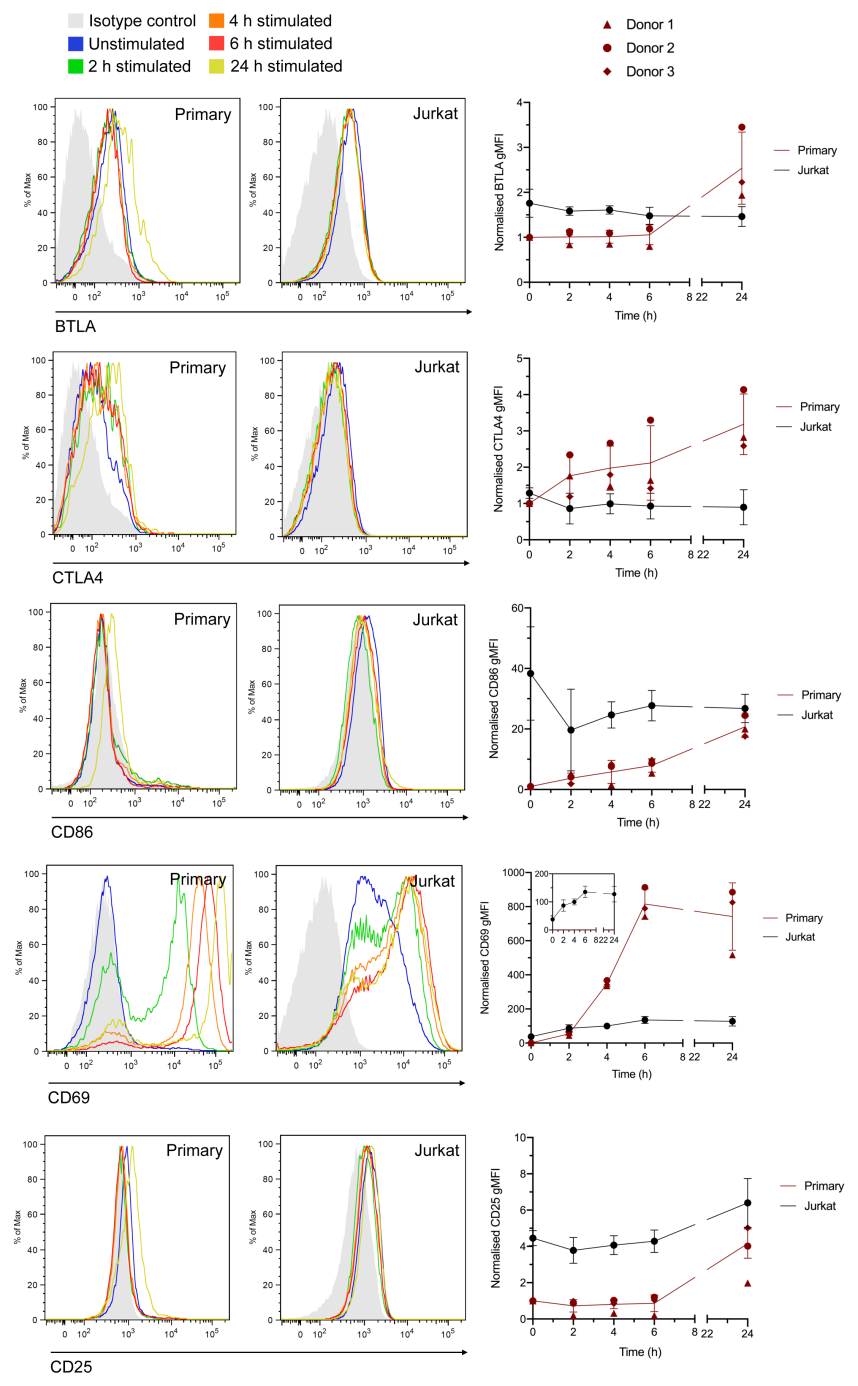
Measurement of stimulation-dependent surface markers using flow cytometry corroborates RNA-Seq data. Histograms (left) and normalised geometric mean fluorescence intensity (gMFI) values (right) for Jurkat and total primary CD4+ T cells stained with anti-BTLA, anti-CTLA4, anti-CD86, anti-CD69, or anti-CD25 antibodies in the resting state or following incubation for 2–24 h. gMFI values are the mean ± std. dev. of 3 independent experiments with 3 different cell donors. Values are normalised to the raw value for resting primary T cells in each experiment (set at 1). Histograms are representative plots from one donor.

Some of the differences between the transcriptional responses of the two cell types is likely due to the detection of genes restricted to the memory CD4 ^+^ lineage but not to effector CD4 ^+^ cells, or may result from Th17 polarisation. Nonetheless, the prevalence of so many known activation-associated genes in the primary-only subset indicates a genuine disparity in transcriptional responses between Jurkat and primary T cells. To test this, Jurkat and total primary CD4 ^+^ T cells were stimulated in the same way as for the RNA-Seq experiments for 2, 4, 6, and 24 h, stained with conjugated antibodies against CD69 and CD25 (which exhibited mRNA upregulation in both cell types), and BTLA, CTLA4, and CD86 (which exhibited mRNA upregulation only in the primary cell data) then assessed by flow cytometry. All five proteins exhibited detectable upregulation following 24 h stimulation in primary cells (with increases in CTLA4, CD86, and CD69 detectable following 2 h stimulation), whereas Jurkats only demonstrated increased expression of CD69 and CD25, in line with the RNA-Seq data ( Figure 4). This indicates that although there is probably some skewing of the primary cell RNA-Seq data towards the memory and Th17 phenotypes, many of the observed differences with Jurkat transcriptional responses are likely to also hold true in conventional CD4 ^+^ T cells under non-polarising conditions.

Although no comparable public datasets exist for non-memory primary CD4 ^+^ T cells activated for 2 h, data are available for total CD4 ^+^ T cells following 24 h activation: GEO: GSE122735 ( Lucic *et al.,* 2019). This allowed comparison with the same RNA-seq data derived from memory CD4 ^+^ cells used earlier but only at a later stage of activation. At this time point, there was strong correlation between log2 fold change relative to unstimulated cells across both primary cell types ( Figure 3I). A much greater proportion of up- and downregulated genes were shared between the two cell types than between memory T cells and Jurkats ( Figure 3J). Genes upregulated only in the total but not memory cells were associated largely with cell division, whilst those upregulated only in memory cells related primarily to Th17 differentiation due to the polarising conditions present during stimulation ( *Extended data* - Dataset S5 ( Felce, 2020b)). Memory cells, but not total CD4 ^+^ T cells, exhibited downregulation of several genes associated with T cell activation and cell-cell interactions ( *Extended data* - Dataset S5 ( Felce, 2020b)). In all, this indicates that although there are differences between the memory and total CD4 ^+^ T cell responses, the majority of observed transcriptional changes correlate across the two cell types. This lends confidence that the comparison described above for Jurkats and memory CD4 ^+^ cells at 2 h stimulation is not inappropriate. This is supported by the observation that there was comparably poor correlation between the 2 h activated Jurkat dataset and both the total and memory primary CD4 ^+^ T cells activated for 24 h, whereas there was much greater correlation between the 2 h and 24 h activated memory ( Figure 6).

### Differences exist between baseline transcriptome in Jurkats and primary CD4 ^+^ cells

**Figure 5.**
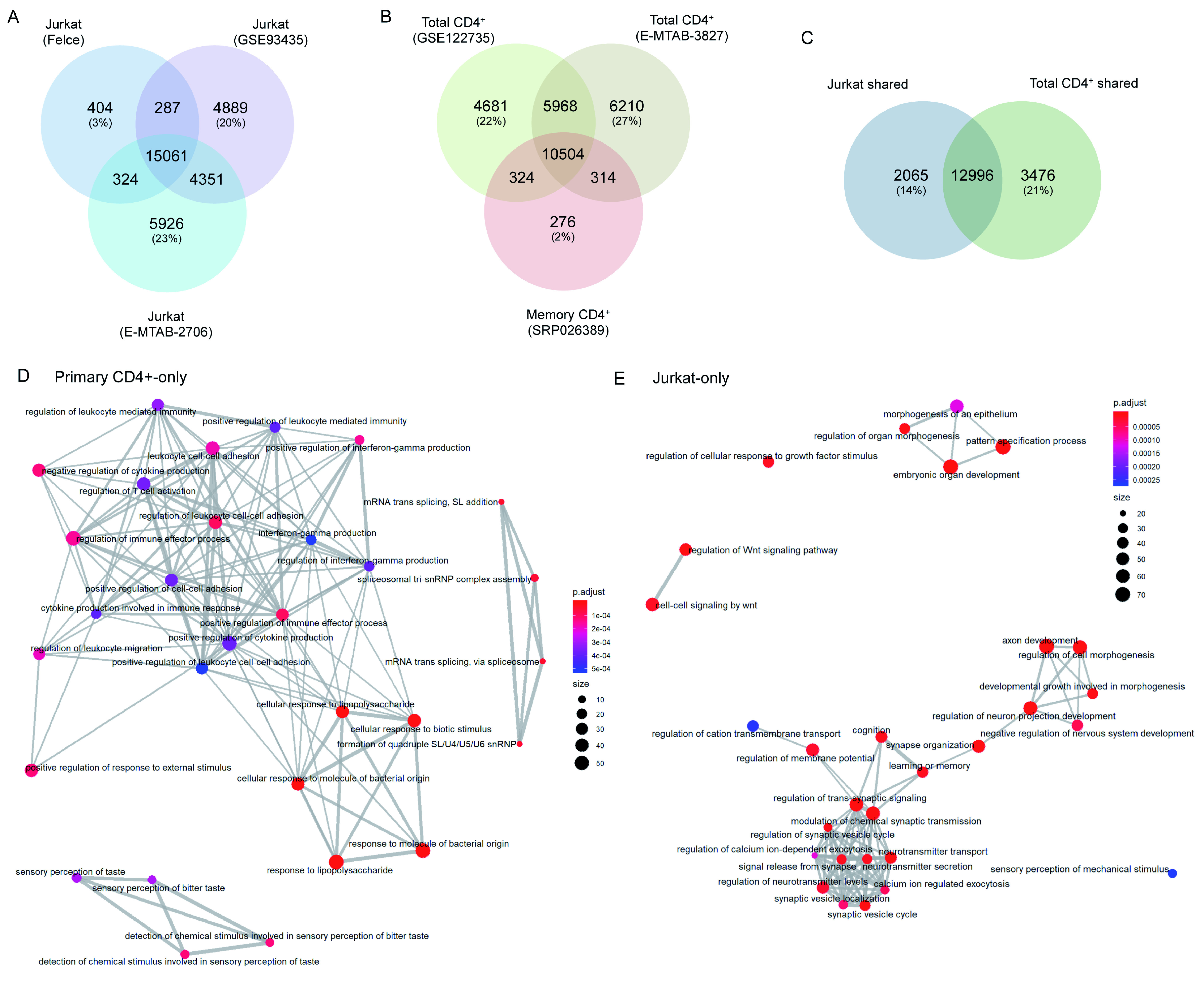
Differences exist between baseline transcriptomes of Jurkat and primary T cells. ( **A**) Venn diagram of total genes expressed in Jurkats under resting conditions in our data (‘Felce’) and two publicly deposited datasets. ( **B**) Venn diagram of total genes expressed in two publicly deposited datasets for resting total primary CD4 ^+^ T cells, and one for resting memory primary CD4 ^+^ T cells. ( **C**) Venn diagram of total genes shared among all three datasets for Jurkats, and both datasets for total primary CD4 ^+^ T cells. ( **D** and **E**) Enrichment map plots of Gene Ontology terms significantly enriched among genes expressed in resting Jurkat or primary CD4 ^+^ T cells, but not both.

In order to confirm the Jurkat line from which we derived these data was not abnormal relative to other Jurkat E6.1 cells in general use, we compared the baseline (i.e. unstimulated) transcriptome of our cells to two publicly available Jurkat RNAseq datasets: Expression Atlas: E-MTAB-2706 ( Klijn *et al.,* 2015) and GEO: GSE93435 ( ENCODE Project Consortium, 2012). Over 93% of genes with baseline expression (mean FKBP > 0.1) in our Jurkats were also expressed in both other samples, whereas only 3% of genes were unique to our Jurkats ( Figure 5A). We are therefore confident that our cells are representative of generally used Jurkat E6.1 lines. General variability across the other Jurkat datasets was ~20% unique genes/sample. Comparable levels of variability were observed when comparing datasets for baseline transcription in primary CD4+ T cells: GSE122735 ( Lucic *et al.,* 2019; as used earlier); and Expression Atlas: E-MTAB-3827 ( Figure 5B). Baseline transcription in the CCR6 ^+^ memory T cell dataset used in the earlier comparisons (SRP026389) was almost entirely (98%) shared with at least one total CD4 ^+^ T cell dataset ( Figure 5B).

**Figure 6.**
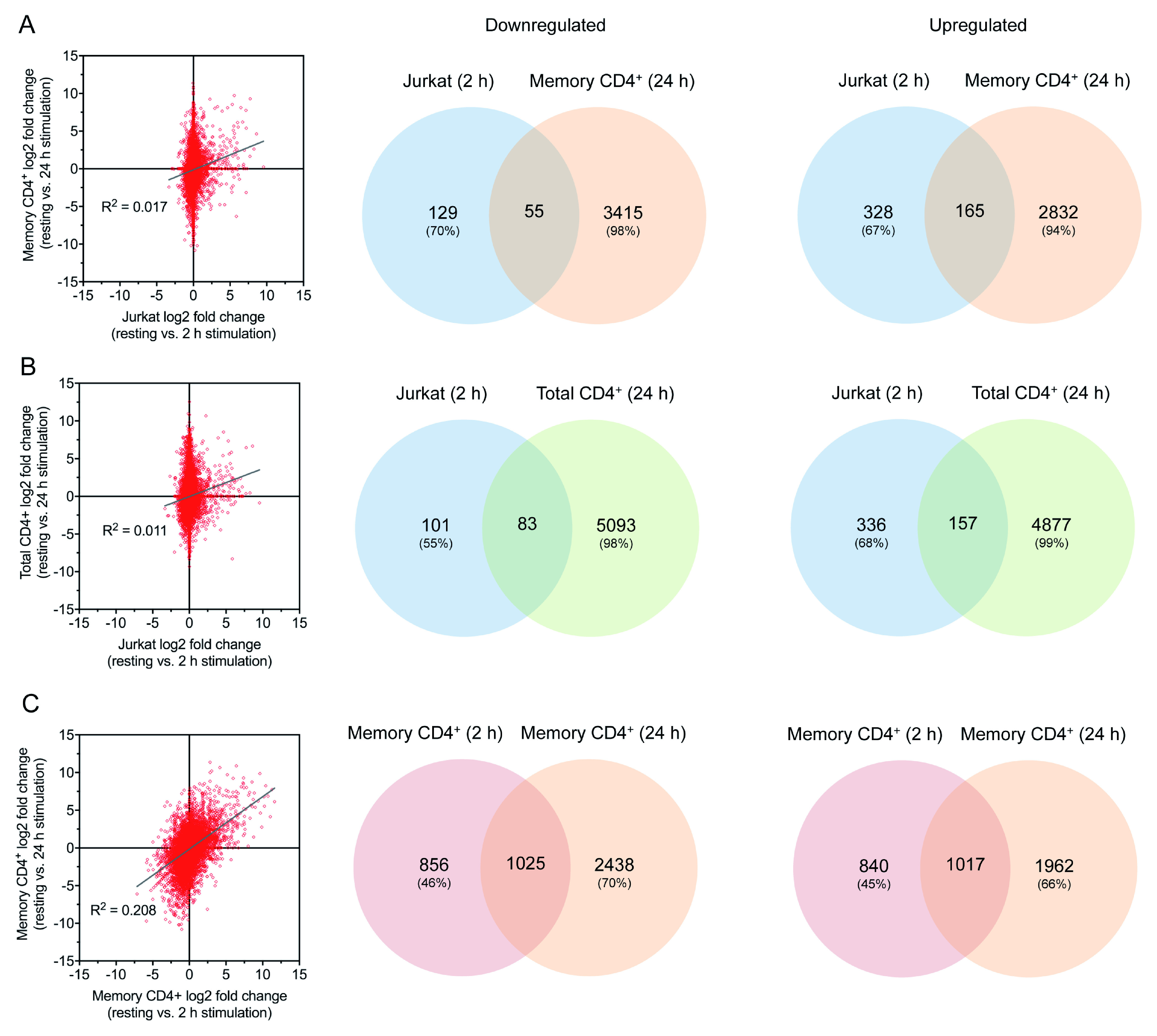
Comparisons of 2 h and 24 h stimulated samples. ( **A**– **C**) Comparison of gene log2 fold change (left) and Venn diagrams for up- and downregulated genes (right) for 2 h stimulated Jurkats vs. 24 h stimulated memory CD4 ^+^ T cells ( **A**), 2 h stimulated Jurkats vs. 24 h stimulated total CD4 ^+^ T cells ( **B**), and 2 h stimulated vs. 24 h stimulated memory CD4 ^+^ T cells ( **C**).

We took this opportunity to broadly compare the baseline transcriptomes of Jurkat and primary CD4 ^+^ T cells. In total, 15,061 genes were shared between our and both public Jurkat datasets, while 16,472 genes were shared between both total primary CD4 ^+^ T cell datasets. The majority of these genes were shared across both cell types, however both also exhibited substantial fractions of genes that were cell type-specific (14% in Jurkats, 21% in primary CD4 ^+^ cells; Figure 5C). Many genes expressed in primary CD4 ^+^ T cells but not Jurkats associated closely with several GO terms connected to T cell responses; predominantly innate immune activation, cytokine secretion, and leukocyte adhesion/migration ( Table 2, Figure 5D; *Extended data* - Dataset S6 ( Felce, 2020b)). By contrast, Jurkat-restricted genes did not generally associate with immune function but instead with a range of developmental and/or neurological biological processes ( Table 2, Figure 5E; *Extended data* - Dataset S6 ( Felce, 2020b)). This again indicates that Jurkats do not fully reproduce the transcriptional state of primary T cells, and hence there may be deviations in the activation-induced transcriptional changes between the two cell types.

**Table 2.** Gene Ontology (GO) terms associated with unique baseline transcriptomes of resting Jurkat and primary T cells. Top 20 GO terms with greatest enrichment in genes shared between multiple datasets for Jurkats or primary CD4 ^+^ T cells but not between the two cell types. Redundant terms have been removed for clarity. A full list of significant terms is available in the Extended Data (Dataset S6).

Cell type	GO Term	Description	P-value
Primary CD4+	GO:0002237	Response to molecule of bacterial origin	2.58E-07
	GO:0032496	Response to lipopolysaccharide	2.89E-07
	GO:0071216	Cellular response to biotic stimulus	1.83E-06
	GO:0000353	Formation of quadruple SL/U4/U5/U6 snRNP	3.48E-05
	GO:0000365	mRNA trans splicing, via spliceosome	3.48E-05
	GO:0002699	Positive regulation of immune effector process	0.0001
	GO:1903037	Regulation of leukocyte cell-cell adhesion	0.0001
	GO:0050912	Detection of chemical stimulus involved in sensory perception of taste	0.000123
	GO:0032103	Positive regulation of response to external stimulus	0.000123
	GO:0001818	Negative regulation of cytokine production	0.000126
	GO:0032729	Positive regulation of interferon-gamma production	0.000153
	GO:0002685	Regulation of leukocyte migration	0.000189
	GO:0002703	Regulation of leukocyte mediated immunity	0.00032
	GO:0050863	Regulation of T cell activation	0.000364
	GO:0046651	Lymphocyte proliferation	0.000779
	GO:0070673	Response to interleukin-18	0.001427
	GO:0034341	Response to interferon-gamma	0.001427
	GO:0050702	Interleukin-1 beta secretion	0.001499
	GO:0031349	Positive regulation of defense response	0.002356
	GO:0032635	Interleukin-6 production	0.002427
Jurkat	GO:0007389	Pattern specification process	1.31E-13
	GO:0048568	Embryonic organ development	5.41E-11
	GO:0061564	Axon development	1.10E-08
	GO:0050804	Modulation of chemical synaptic transmission	2.01E-07
	GO:0099177	Regulation of trans-synaptic signaling	2.02E-07
	GO:0030111	Regulation of Wnt signaling pathway	4.48E-07
	GO:0010975	Regulation of neuron projection development	5.17E-07
	GO:0099504	Synaptic vesicle cycle	5.17E-07
	GO:0050808	Synapse organization	7.92E-07
	GO:0022604	Regulation of cell morphogenesis	7.92E-07
	GO:0006836	Neurotransmitter transport	7.92E-07
	GO:0050890	Cognition	7.37E-06
	GO:0007611	Learning or memory	9.88E-06
	GO:0090287	Regulation of cellular response to growth factor stimulus	1.21E-05
	GO:0042391	Regulation of membrane potential	1.86E-05
	GO:0051961	Negative regulation of nervous system development	4.24E-05
	GO:0017156	Calcium ion regulated exocytosis	4.87E-05
	GO:0017158	Regulation of calcium ion-dependent exocytosis	0.000108
	GO:0002009	Morphogenesis of an epithelium	0.000114
	GO:1904062	Regulation of cation transmembrane transport	0.000259

## Discussion

The Jurkat E6.1 cell line has been extensively used as a model of T cell biology, particularly in the study of TCR signalling. Here we have presented RNA-Seq data revealing the early transcriptional effects of Jurkat activation through engagement of the TCR and CD28. We have observed that transcriptional modulation following 2 h of stimulation correlates closely with many of the known responses to activation in T cells, and that this is unaffected by the presence of the chemokines CXCL12 and CCL19. Nonetheless, when compared to publicly available data for primary T cells stimulated in the same manner it is evident that these changes represent only a small fraction of the early responses occurring in primary cells. Many genes lacking modulation in Jurkats are closely associated with known pathways of T cell activation, and a subset of these was validated at the protein level using flow cytometry. This is underlined further by substantial differences in the baseline, resting transcriptomes of Jurkat and primary T cells.

The observation that Jurkat E6.1 cells do not fully replicate the early transcriptional modulation of primary T cells is perhaps not surprising given the known differences between Jurkat and primary responses to activation ( Bartelt *et al.,* 2009; Colin-York *et al.,* 2019; Shan *et al.,* 2000); however, the extent of this difference is quite striking. Differences in the transcriptional responses of Jurkats and peripheral blood mononuclear cells exposed to mycotoxin have been reported previously ( Katika *et al.,* 2012), though in this case the heterogenous nature of the primary cell sample complicates interpretation. Similarly, there are several caveats to the interpretation of the comparisons made here that must be considered. Most obvious is the fact that Jurkats are not memory T cells, yet the dataset used for direct comparison at the 2 h timepoint was collected from CCR6 ^+^ CD4 ^+^ memory T cells. As a result, many memory-specific responses are likely to differ, such as upregulation of costimulatory ligands. Furthermore, the activation conditions were not identical in both cases due to the presence of Th17-polarising chemokines with the primary T cells, explaining the upregulation of several Th17-associated genes (e.g. *IL17F*) in the primary sample. Nonetheless, the core activatory signals were the same in both cases, and so it seems reasonable to expect that many central transcriptional responses should be conserved between both cell types. The lack of evident transcriptional regulation in Jurkats for a majority of genes altered in primary cells indicates a fundamental difference between the two cell types, in which the Jurkat transcriptome is much less responsive to early activatory signals.

It must also be considered that the primary memory CD4 ^+^ T cell data were derived from a single donor, which we are assuming is representative of normal primary T cell behaviour. There is some reassurance that this is the case from the 24 h stimulation condition, which exhibits good correlation with data derived from multiple donors of total CD4 ^+^ T cells ( Figure 3I); however, a donor-specific effect cannot be ruled out.

An alternative explanation for these observations is that transcriptional modulation in Jurkats is slower than in primary cells, and hence many apparently primary-specific changes in gene expression may be replicated in Jurkats at later times post-stimulation. There does not appear to be a general trend among typical T cell activation markers to exhibit greater log2 fold change in the primary cell data (e.g. *CD69* 3.71 in Jurkats vs. 7.54 in primary cells; *IL2* 6.71 vs. 9.38; *IL2RA* 5.44 vs. 2.74; *IFNG* 7.74 vs. 7.39), and for the proteins assessed directly by flow cytometry this also does not appear to be the case ( Figure 4). Nonetheless, this cannot be fully excluded as a general effect.

In summary, these data indicate that, at least at a transcriptional level, Jurkat E6.1 cell responses are far more minimal than those in primary T cells, and hence Jurkats represent a highly simplified model of T cell transcriptional modulation. We fully acknowledge the limitations of the comparisons made, insofar as the differences existing between cell types and activation conditions; however, the extent of the observed transcriptional differences and the direct validation of several key examples lends confidence to the central conclusions reported here. We provide the present dataset for other researchers to use in more direct comparisons to test the robustness of our conclusions.

## Data availability

### Underlying data

Gene Expression Omnibus: RNA-Seq of resting and activated Jurkat E6.1 cells. Accession number GSE145453; https://identifiers.org/geo:GSE145453.

Open Science Framework: Flow cytometry data of Jurkat and Primary CD4+ cells post stimulation. http://doi.org/10.17605/OSF.IO/HAXMY ( Felce, 2020a).

This project contains the following underlying flow cytometry data:

- E1 – Flow cytometry data form donor 1.
- E2 – Flow cytometry data form donor 2.
- E3 – Flow cytometry data form donor 3.

### Extended data

Open Science Framework: RNA-Seq analysis of early transcriptional responses to activation in the leukaemic Jurkat E6.1 T cell line. http://doi.org/10.17605/OSF.IO/7X8CG ( Felce, 2020b).

This project contains the following extended data:

- Dataset S1 – Raw count values for all samples.
- Dataset S2 – Full list of differentially expressed genes between resting and 2 h stimulated (no chemokine) conditions.
- Dataset S3 – Full list of GO terms significantly associated with differentially expressed genes between resting and 2 h stimulated (no chemokine) conditions.
- Dataset S4 – Full list of GO terms significantly associated with differentially expressed genes shared and non-shared between Jurkat and primary memory CD4 ^+^ T cells stimulated for 2 h.
- Dataset S5 – Full list of GO terms significantly associated with differentially expressed genes shared and non-shared between primary total CD4 ^+^ and primary memory CD4 ^+^ T cells stimulated for 24 h.
- Dataset S6 – Full list of GO terms significantly associated with genes expressed under resting conditions that are unique to Jurkat or total primary CD4 ^+^ T cells.

Data are available under the terms of the Creative Commons Attribution 4.0 International license (CC-BY 4.0).
