# Supplementary material for: RNA-Seq analysis of early transcriptional responses to activation in the leukaemic Jurkat E6.1 T cell line
Source: Wellcome Open Res. 2021 Jun 17;5:42. Originally published 2020 Mar 3. [Version 2] doi: 10.12688/wellcomeopenres.15748.2 (PMC9971649; doi:10.12688/wellcomeopenres.15748.2)
Supplement: Peer review history

# Peer review: Reviewer response for version 2

**DOI:** 10.21956/wellcomeopenres.18671.r53925

**Published:** 2023-01-30

**Authors:** İpek Erdoğan Vatansever

**Affiliations:** 1 Department of Molecular Biology and Genetics, Noncoding RNA Laboratory, Faculty of Science, Izmir Institute of Technology, Izmir, Turkey

This manuscript discusses whether Jurkat cells, human acute lymphoblastic leukemia T-cell line, are a suitable model for studies of T cell receptor response. In this context, as explained in detail after the revisions, the data obtained from cells that were either unstimulated or stimulated with CD3/CD28 beads for 2 hours was compared to the datasets of activated different T cell subsets. This study, in which the adequacy of the Jurkat cell model is discussed through both RNA sequencing analyzes and flow cytometry data, has gained scientific competence, reproducibility and originality in its field, with revisions made in the light of the criticisms previously stated by other reviewers. In particular, comprehensive comparison of RNA sequencing data will serve as a guide for other research groups in this field.

Is the work clearly and accurately presented and does it cite the current literature?

Yes

If applicable, is the statistical analysis and its interpretation appropriate?

I cannot comment. A qualified statistician is required.

Are all the source data underlying the results available to ensure full reproducibility?

Yes

Is the study design appropriate and is the work technically sound?

Yes

Are the conclusions drawn adequately supported by the results?

Yes

Are sufficient details of methods and analysis provided to allow replication by others?

Yes

Reviewer Expertise:

Non-coding RNA, programmed cell death

I confirm that I have read this submission and believe that I have an appropriate level of expertise to confirm that it is of an acceptable scientific standard.

# Peer review: Reviewer response for version 2

**DOI:** 10.21956/wellcomeopenres.18671.r44525

**Published:** 2021-07-27

**Authors:** Scott McComb

**Affiliations:** 1 Human Health Therapeutics Research Centre, National Research Council Canada, Ottawa, Canada; 2 The University of Ottawa Centre for Infection, Immunity, and Inflammation, Ottawa, Canada; 3 Department of Biochemistry, Microbiology, and Immunology, Faculty of Medicine, University of Ottawa, Ottawa, Canada

In the revised manuscript Felce *et al.* have added new analysis comparing resting Jurkat cell transcriptomic data to publicly available single cell sequencing data for CD4 T cells. This data rounds out the study well, and adds to the robustness of this analysis. It would be helpful to see the activated Jurkat profile included in this comparison to see whether activated Jurkat cells might even more closely align with proliferating CD4+ T cells. 

 As a minor criticism, it would be preferable to see gene names used in Figure 1E to enhance the readability of this sub-figure. 

 Overall, this study remains a helpful and relatively robust analysis of Jurkat transcriptomics, providing good molecular evidence to support the dogma within the immunology field that Jurkat cells can serve as a model of early T cell receptor signaling and activation but poorly recapitulate the complexities of later T cell activation, differentiation, and function.

Is the work clearly and accurately presented and does it cite the current literature?

Partly

If applicable, is the statistical analysis and its interpretation appropriate?

I cannot comment. A qualified statistician is required.

Are all the source data underlying the results available to ensure full reproducibility?

Yes

Is the study design appropriate and is the work technically sound?

Yes

Are the conclusions drawn adequately supported by the results?

Yes

Are sufficient details of methods and analysis provided to allow replication by others?

Yes

Reviewer Expertise:

Cancer immunology, leukemia, programmed cell death, synthetic immmunology.

I confirm that I have read this submission and believe that I have an appropriate level of expertise to confirm that it is of an acceptable scientific standard.

# Peer review: Reviewer response for version 1

**DOI:** 10.21956/wellcomeopenres.17267.r41089

**Published:** 2020-11-10

**Authors:** Scott McComb

**Affiliations:** 1 Human Health Therapeutics Research Centre, National Research Council Canada, Ottawa, Canada; 2 The University of Ottawa Centre for Infection, Immunity, and Inflammation, Ottawa, Canada; 3 Department of Biochemistry, Microbiology, and Immunology, Faculty of Medicine, University of Ottawa, Ottawa, Canada

In the manuscript entitled "RNA-Seq analysis of early transcriptional responses to activation in the leukaemic Jurkat E6.1 T cell line", Felce *et al.* have generated whole-transcriptome RNA-sequencing data for Jurkat E6.1 cells with and without stimulation using CD3/CD28 bead stimulation for 2 hours. In addition to stimulation with CD3/CD28 beads, authors also tested the effect of chemokine stimulation.

 Using the Jurkat-activation RNA-seq dataset described above, authors make extensive comparisons to a published dataset of activated primary CCR6 ^+^ CD4 ^+^ memory T cells (Zhao *et al*., 2014); finding only limited correlation between the transcriptional responses observed in Jurkat cells and memory CD4+ T cells. Authors then confirm divergence in a small number of surface markers using flow analysis of activated Jurkat or primary CD4+ T cells at various timepoints. 

 The main findings of the paper in comparing Jurkat dataset with that of stimulated memory CD4 or total CD4 cells (for 24h) are valid and interesting. Broadly, these findings would be consistent with the view in the field that Jurkat cells are capable of activating some components of the early T cell activation pathway in response to CD3/CD28 stimulation, but Jurkat activation is radically less complex than that seen in genuine T cells. The dataset generated here provides strong data to support that view, and contributes significantly to literature on Jurkat cells. 

 Critiques: 

1. Some of the specific choices made for activation seem quite arbitrary. The justification for choosing 2 hours post stimulation seems somewhat weak for instance. It would have been interesting to see more timepoints investigated via RNA-seq. 
2. The addition of chemokine stimulation in initial Jurkat stimulation seems to result in limited insight was gained through the addition of chemokine stimulation and no comparator primary T cell chemokine-stimulated dataset was investigated. Authors should either further investigate to compare primary and Jurkat data with chemokines and discuss or move chemokine data to supplemental data to streamline and focus the manuscript on key findings.
3. The validity of CD4+ memory cells as a comparator of Jurkat cells is questionable. This specific memory CD4+ subset may reflect a very specific subtype of T cells. While there may be limited datasets available for RNA-sequencing, could some comparison not be made with the plethora of microarray data available for activated T cells? (see Immgen.org). Investigating comparison with single cell RNA-seq to identify which T cell type most closely aligns with Jurkat cells might be an interesting question to probe as well.

Overall, I see no major scientific oversights in this publication. The data is novel and interesting and this reviewer would recommend that the manuscript should be accepted with minor revision to address concerns discussed above.

Is the work clearly and accurately presented and does it cite the current literature?

Partly

If applicable, is the statistical analysis and its interpretation appropriate?

I cannot comment. A qualified statistician is required.

Are all the source data underlying the results available to ensure full reproducibility?

Yes

Is the study design appropriate and is the work technically sound?

Yes

Are the conclusions drawn adequately supported by the results?

Yes

Are sufficient details of methods and analysis provided to allow replication by others?

Yes

Reviewer Expertise:

Cancer immunology, leukemia, programmed cell death, synthetic immmunology.

I confirm that I have read this submission and believe that I have an appropriate level of expertise to confirm that it is of an acceptable scientific standard, however I have significant reservations, as outlined above.

# Response

**Published:** 2021-06-06

**Authors:** James Felce

**Affiliations:** University of Oxford, UK

We thank the reviewer for their detailed assessment of our work and useful feedback. We have made the following amendments in relation to the following specific points:

*Some of the specific choices made for activation seem quite arbitrary. The justification for choosing 2 hours post stimulation seems somewhat weak for instance. It would have been interesting to see more timepoints investigated via RNA-seq.*

 We agree that it would be interesting to undertake a more thorough investigation of the kinetics of Jurkat transcriptional responses relative to primary cells. The original justification for the chosen two-hour timepoint was due to the need to focus on very early transcriptional responses in order to inform other work that was being undertaken to examine the architecture of the immunological synapse and how transcriptional changes may influence this. Unfortunately, collecting further RNA-Seq data at later timepoints is not possible within the scope of this project as the original funding for the project has been exhausted and the key authors have moved positions. We nonetheless think that the data collected at this early timepoint is still of use to the field.

*The addition of chemokine stimulation in initial Jurkat stimulation seems to result in limited insight was gained through the addition of chemokine stimulation and no comparator primary T cell chemokine-stimulated dataset was investigated. Authors should either further investigate to compare primary and Jurkat data with chemokines and discuss or move chemokine data to supplemental data to streamline and focus the manuscript on key findings.*

 This is a valid point. We have included these data primarily to act as a resource for others considering similar work, even though they do not advance the key conclusions of the study. Unfortunately the format of WOR does not permit supplemental material otherwise we would move these data out of the main manuscript. Even though they do not provide substantial insight, we believe that these data are worthy of inclusion as negative results.

*The validity of CD4+ memory cells as a comparator of Jurkat cells is questionable. This specific memory CD4+ subset may reflect a very specific subtype of T cells. While there may be limited datasets available for RNA-sequencing, could some comparison not be made with the plethora of microarray data available for activated T cells? (see Immgen.org).*

 We have unfortunately not been able to find suitable public microarray data that match the activation conditions in human cells. The publications that we have found with similar conditions do not appear to have publicly deposited raw data (possibly due to their age), and the published gene lists are not in formats that we can process. However, please see the point below regarding single cell RNA-Seq as an alternative to determine the relative difference between Jurkats and different primary CD4 ^+^ subtypes.

*Investigating comparison with single cell RNA-seq to identify which T cell type most closely aligns with Jurkat cells might be an interesting question to probe as well.*

 We thank the reviewer for this interesting suggestion. We have now included a comparison between our resting Jurkat data and public single cell RNA-Seq data of different primary CD4 ^+^ subsets, including both effector and central memory cells. This analysis revealed that the primary cells were broadly comparable in their difference from the Jurkat transcriptome, with the exception of proliferating CD4 ^+^ cells. This is presumably due to the activation of genes involved in cell cycle progression in both Jurkats and proliferating primary cells. The differences between primary cell populations was much smaller than those between primary and Jurkat, further reinforcing the notion that Jurkats possess a highly abnormal transcriptional profile. The fact that the difference between the primary CD4 ^+^ subtypes is small compared to the difference between primary CD4 ^+^ and Jurkats validates the use of memory CD4 ^+^ T cell subsets in comparisons.

# Peer review: Reviewer response for version 1

**DOI:** 10.21956/wellcomeopenres.17267.r38097

**Published:** 2020-04-06

**Authors:** Oxana Dobrovinskaya

**Affiliations:** 1 Laboratory of Immunobiology and Ionic Transport Regulation, University Center for Biomedical Research, University of Colima, Colima, Mexico

Jurkat cells are leukemic lymphoblasts derived from the blood of young patient diagnosed with acute lymphoblastic leukaemia of T lineage (T-ALL). These leukemic cells possess a T cell receptor (TCR) which is capable of mounting biologically relevant responses to TCR stimulation, so many details of signal transduction during T cell activation were discovered with use of this cell line. However, despite the ability to be activated via TCR, Jurkat are not healthy but transformed leukemic cells. Therefore, this model has certain limitations that should be considered if you nevertheless decided to use it in your experiments. Present work was designed in order to demonstrate how reasonable is the use of Jurkat leukemic lymphoblastic cell line as the model to study the process of T cell activation in response to T cell receptor (TCR) engagement. The work was carried out by 4 researchers from UK working in the field of T cell biology and immunological synapsis. The question was addressed previously (for reference, see for example Bartelt *et al*., 2009) ^1^ where the differences in signalling events were revealed between leukemic cell lines and healthy activated cells from peripheral blood. The authors of the present study addressed an important and relevant questions about differences in the processes of activation in Jurkat and healthy primary T cells at transcriptional level. They present whole-transcriptome RNA-Sequencing data for Jurkat E6.1 cells in the resting state and during early activation response (antiCD3/CD28), in the presence and absence of the chemokines CXCL12 and CCL19, and also perform a basic comparison between transcriptional responses in Jurkat E6.1 cells and those in primary human T lymphocytes. The authors concluded that Jurkat E6.1 cells represent a simplified model of early T cell transcriptional responses.

 I consider that this work is highly useful for all researchers working in the field of T cell biology, both with healthy T cells and leukaemia of T lineage. I have some observations regarding article presentation: 

1. Taking into account that behaviour characteristics (proliferation rate, population doubling time, etc) and the pattern of gene expression may depend on passage number, the corresponding information (range of passage numbers) should be indicated in the Materials and methods section, to enable other researchers to reproduce the data of this study.
2. It should be noted here that leukemic cell lines are frequently used not as general T cell model but rather as T-ALL model, in particular in the field of drug and drug targets discovery, etc. However, being derived from different patients and representing transformed T cells at different maturation stages, with different phenotypes and mutations patterns, neither Jurkat nor any other leukemic T cell line could be considered as a typical leukemic model. Therefore, comparative characterization of leukemic cell lines is emergently necessary. Instead of use of “gold standard” or “typical” leukemic cell line (Jurkat, or any other cell line) in these kind of studies, the use of several cell lines should be strongly recommended. Although optional, I would recommend to mention and clarify this point in Discussion section.
3. Please provide an explanation for your result that Jurkat-restricted genes did associate with “neurological biological processes”. 

Is the work clearly and accurately presented and does it cite the current literature?

Yes

If applicable, is the statistical analysis and its interpretation appropriate?

Yes

Are all the source data underlying the results available to ensure full reproducibility?

Yes

Is the study design appropriate and is the work technically sound?

Yes

Are the conclusions drawn adequately supported by the results?

Yes

Are sufficient details of methods and analysis provided to allow replication by others?

Partly

Reviewer Expertise:

Signal transduction pathways in T-ALL, antileukemic drugs.

I confirm that I have read this submission and believe that I have an appropriate level of expertise to confirm that it is of an acceptable scientific standard, however I have significant reservations, as outlined above.

## References

[1] : Comparison of T cell receptor-induced proximal signaling and downstream functions in immortalized and primary T cells. *PLoS One* .2009;4(5) : 10.1371/journal.pone.0005430 e5430 10.1371/journal.pone.0005430 19412549PMC2673025

# Response

**Published:** 2021-06-06

**Authors:** James Felce

**Affiliations:** University of Oxford, UK

We thank the reviewer for their detailed assessment of our work and useful feedback. We have made the following amendments in relation to the following specific points:

*Taking into account that behaviour characteristics (proliferation rate, population doubling time, etc) and the pattern of gene expression may depend on passage number, the corresponding information (range of passage numbers) should be indicated in the Materials and methods section, to enable other researchers to reproduce the data of this study.*

 Passage numbers have now been added to the Methods section.

*It should be noted here that leukemic cell lines are frequently used not as general T cell model but rather as T-ALL model, in particular in the field of drug and drug targets discovery, etc. However, being derived from different patients and representing transformed T cells at different maturation stages, with different phenotypes and mutations patterns, neither Jurkat nor any other leukemic T cell line could be considered as a typical leukemic model. Therefore, comparative characterization of leukemic cell lines is emergently necessary. Instead of use of “gold standard” or “typical” leukemic cell line (Jurkat, or any other cell line) in these kind of studies, the use of several cell lines should be strongly recommended. Although optional, I would recommend to mention and clarify this point in Discussion section.*

 We agree that this is an interesting point. This has now been added to the Discussion.

*Please provide an explanation for your result that Jurkat-restricted genes did associate with “neurological biological processes”.*

 Many of the Jurkat-enriched genes related to the neuronal-associated GO terms are also linked with several other GO terms, and are not highly neurone-specific. These include many genes involved in broad signal transduction mechanisms – including semaphorins, Wnt molecules, GPCRs, and Ephrins/Ephrin receptors. Many of these are known to be involved in immune processes (e.g. EPHB2: https://www.frontiersin.org/articles/10.3389/fimmu.2019.01473/full). Similarly, a number of transcription factors are also found in this dataset, and several are known to be expressed in T cells (e.g. FOXO6: https://www.ncbi.nlm.nih.gov/pmc/articles/PMC3053075/). The association of Jurkat-restricted genes with these terms almost certainly does not represent neuronal-specific differentiation of the cells, but rather that these genes have broad functionality and are also annotated as involved in neurological processes alongside their immune functions.
